# Epidemiological characterisation of the first 785 SARS-CoV-2 Omicron variant cases in Denmark, December 2021

**DOI:** 10.2807/1560-7917.ES.2021.26.50.2101146

**Published:** 2021-12-16

**Authors:** Laura Espenhain, Tjede Funk, Maria Overvad, Sofie Marie Edslev, Jannik Fonager, Anna Cäcilia Ingham, Morten Rasmussen, Sarah Leth Madsen, Caroline Hjorth Espersen, Raphael N. Sieber, Marc Stegger, Vithiagaran Gunalan, Bartlomiej Wilkowski, Nicolai Balle Larsen, Rebecca Legarth, Arieh Sierra Cohen, Finn Nielsen, Janni Uyen Hoa Lam, Kjetil Erdogan Lavik, Marianne Karakis, Katja Spiess, Ellinor Marving, Christian Nielsen, Christina Wiid Svarrer, Jonas Bybjerg-Grauholm, Stefan Schytte Olsen, Anders Jensen, Tyra Grove Krause, Luise Müller

**Affiliations:** 1 Infectious Disease Epidemiology and Prevention, Statens Serum Institut, Copenhagen, Denmark; 2 European Programme for Intervention Epidemiology Training (EPIET), European Centre for Disease Prevention and Control, (ECDC), Stockholm, Sweden; 3 Department of Bacteria, Parasites and Fungi, Statens Serum Institut, Copenhagen, Denmark; 4 Virus and Microbiological Special Diagnostics, Statens Serum Institut, Copenhagen, Denmark; 5 COVID-19 tracing Unit, Danish Patient Safety Authority, Copenhagen, Denmark; 6 TestCenter Denmark, Statens Serum Institut, Copenhagen, Denmark; 7 Danish National Biobank, Statens Serum Institut, Copenhagen, Denmark; 8 The Data integration and Analysis Secretariat, Statens Serum Institut, Copenhagen, Denmark; 9 Congenital disorders, Statens Serum Institut, Copenhagen, Denmark; 10 Epidemiological Infectious Disease Preparedness, Statens Serum Institut, Copenhagen, Denmark

**Keywords:** COVID-19, Omicron, surveillance, epidemiology, Denmark, viral infections, laboratory, SARS-CoV-2

## Abstract

By 9 December 2021, 785 SARS-CoV-2 Omicron variant cases have been identified in Denmark. Most cases were fully (76%) or booster-vaccinated (7.1%); 34 (4.3%) had a previous SARS-CoV-2 infection. The majority of cases with available information reported symptoms (509/666; 76%) and most were infected in Denmark (588/644; 91%). One in five cases cannot be linked to previous cases, indicating widespread community transmission. Nine cases have been hospitalised, one required intensive care and no deaths have been registered.

On 26 November 2021, the World Health Organization designated the severe acute respiratory syndrome coronavirus 2 (SARS-CoV-2) Omicron variant (Phylogenetic Assignment of Named Global Outbreak (Pango) lineage designation B.1.1.529) a variant of concern [1]. Two days later, on 28 November 2021, the first two Omicron cases were identified in Denmark in travellers returning from South Africa (see detailed timeline in Table 1). By 9 December, a total of 785 Omicron cases have been registered in Denmark. The aim of this communication is to describe these first Danish cases to provide insights into the spread and early indications of severity.

**Table 1:** Timeline of SARS-CoV-2 Omicron developments, Denmark, November and December 2021

Development	Date	Reference
South Africa announces detection of a new SARS-CoV-2 variant B.1.1.529	25 November	[12]
World Health Organization designates B.1.1.529 as a variant of concern, named Omicron	26 November	[1]
Travel restrictions to South Africa and six neighbouring countries^a^ are put in place. This includes: - Advice against travelling to these countries; - For people with recent stays (≤ 10 days) in these countries, testing within 24 h after arrival in Denmark and isolation for 10 days; - Restriction of travel without negative COVID-19 test; - Incoming travellers from these countries are only allowed entry to Denmark with recognisable purpose and when holding a negative COVID-19 testing certificate.	27 November	[13]
First two Omicron cases confirmed by WGS in Denmark in returning travellers from South Africa; Extensive contact tracing for Omicron cases implemented: - Confirmed cases are to isolate until 48 h after symptom cessation - Close contacts as well as close contacts of close contacts are recommended to quarantine regardless of vaccination status and to take a PCR test on Days 1, 4 and 6 from last exposure; recommendation of quarantine until a negative result of the test on Day 6.	28 November	[14,15]
Implementation of travel restrictions from three additional countries (Angola, Malawi and Zambia)	29 November	[16]
All passengers arriving from Hamad International Airport, Qatar and Dubai International Airport, United Arab Emirates, to be tested by antigen test at arrival in Denmark and referred to PCR test if the antigen test is positive; Omicron variant detection changes from del 69–70 to the more specific 452L.	1 December	[17]
Change in the case definition of confirmed Omicron cases from WGS only to variant PCR and/or WGS	4 December	[18]
Quarantine requirement for close contacts of close contacts of Omicron cases is abolished.	7 December	[19]

COVID-19: coronavirus disease; SARS-CoV-2: severe acute respiratory syndrome coronavirus 2; WGS: whole genome sequencing.

**^a^** Botswana, Eswatini, Lesotho, Mozambique, Namibia and Zimbabwe.

## Surveillance of SARS-CoV-2 variants in Denmark

An important pillar in the Danish coronavirus disease (COVID-19) response has been free and easy access to testing. Community testing is carried out in ‘TestCenter Denmark’ (TCDK), where every resident or visitor in Denmark can book an appointment (TCDK carried out more than 85% of all tests [2] and uses reverse transcription PCR (RT-PCR) for SARS-CoV-2 confirmation). Furthermore, testing is also carried out through referral mainly from general practitioners or hospitals as part of the national healthcare testing system, for which samples are analysed in one of 10 local clinical microbiology departments using RT-PCR or other nucleic acid amplification tests. The testing strategy is supplemented by an extensive antigen-testing programme, and persons with a positive antigen test are encouraged to get a confirmatory PCR test.

The Danish COVID-19 testing strategy includes asymptomatic testing for close contacts of confirmed cases and screening tests in certain settings (e.g. workplaces and primary schools). Overall, more than 15% of the Danish population is PCR-tested at least once during a week [3]. By 9 December, 9.2% of the Danish population had tested positive for SARS-CoV-2 since the start of the epidemic.

Surveillance of SARS-CoV-2 variants in Denmark is based on screening of all positive samples with variant-specific RT-PCR and extensive whole-genome sequencing (WGS) efforts. For most of 2021, more than 90% of SARS-CoV-2 isolates were sequenced, although rates have been lower since late autumn because of rising case numbers [4]. The TCDK has a maximum capacity of 15,000 WGS per week.

## Public health response to the Omicron variant

An RT-PCR detecting the Omicron variant using the 452L marker (estimated specificity: 99.99% based on retrospective analysis) [5] was developed and implemented in the TCDK by 1 December. In the 10 local clinical microbiology departments, similar solutions have been set up. All isolates from cases who are found to be RT-PCR-positive for the Omicron variant are subsequently subjected to WGS. More WGS data are needed in order to have a meaningful positive predictive value.

A confirmed Omicron case was defined based on WGS results until 3 December. From 4 December, the definition was extended to also include positive RT-PCR for the variant.

Extensive contact tracing around Omicron cases was initiated and is carried out by the Danish Patient Safety Authority (STPS). This includes self-isolation of cases until 48 h after symptom cessation for confirmed cases or, if asymptomatic, self-isolation for 7 days. It is recommended that all close contacts, as well as their close contacts, regardless of vaccination status, are tested (on Day 1, 4 and 6 after last contact with an Omicron case) and self-quarantine until a negative test result has been received after the final test on Day 6. Self-quarantine of close contacts of close contacts was abolished on 7 December 2021. Restrictions on incoming travellers were introduced on 27 November (Table 1).

## Register-based surveillance

To describe the Omicron cases, we used data from the routine Danish surveillance of COVID-19 [6] in which information from several national registries is linked daily. This provides information on demographic characteristics, vital status, vaccination status, previous SARS-CoV-2 infection, admission to hospital and intensive care treatment of cases. During contact tracing, the STPS collects information on travel history symptoms and suspected place of infection. 

## Characterisation of cases

**Figure 1:**
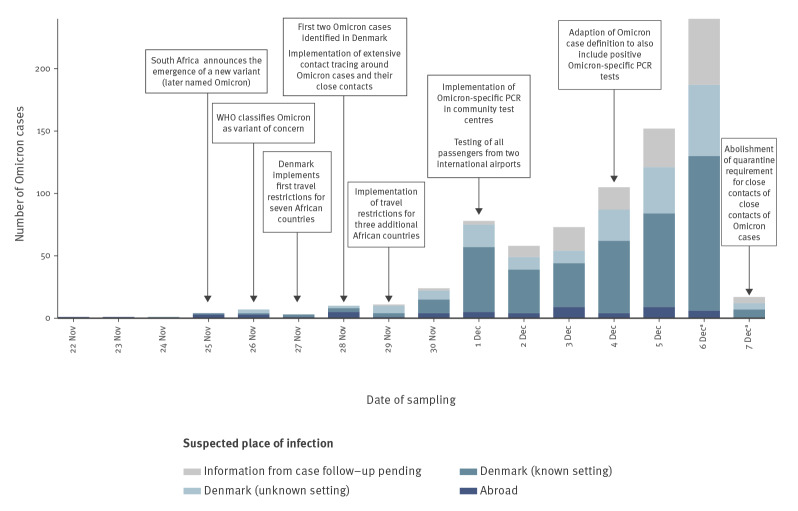
Sample date for SARS-CoV-2 Omicron cases by suspected place of infection, Denmark, 22 November–7 December 2021, data as at 9 December 2021 (n = 785)

COVID-19: coronavirus disease; SARS-CoV-2: severe acute respiratory syndrome coronavirus 2; WGS: whole genome sequencing; WHO: World Health Organization.

^a^ Note that the number of cases from 6 and 7 December is not fully representative as data are pending.

As per 9 December 2021, 785 cases of SARS-CoV-2 Omicron have been registered in Denmark. As there is a few days' lag from sampling to test result, the latest sampling date was 7 December. Daily case counts rose quickly (Figure 1), with daily increases of more than 40% from 4 December. In total, 143 (18%) have so far been confirmed by WGS. The age of cases ranged between 2 and 95 years (median: 32) and 433 (55%) were male (Table 2). Nine cases (1.2%) are or have been hospitalised, one case so far has received intensive care treatment and no cases have died. Cases were identified widespread across the country, but most were clustered in two main epicentres in the western part of Denmark and in the area of the Capital. A total of 599 (76%) cases were fully vaccinated and an additional 56 (7.1%) had received full vaccination plus a booster dose. Number and proportions of cases with the SARS-CoV-2 Delta variant (B.1.617.2 and sublineages) in the same period are also shown in Table 2 for reference.

**Table 2:** Characteristics of SARS-CoV-2 Delta and Omicron variant cases, Denmark, 22 November–7 December 2021

	Number of Delta^a^ cases (n = 19,137)	% of all Delta^a^ cases	Number of Omicron cases (n = 785)	% of all Omicron cases
**Age (years)**				
0–9	3,081	16	30	3.8
10–14	2,434	13	23	2.9
15–19	962	5.0	102	13
20–29	2,317	12	214	27
30–39	2,548	13	110	14
40–49	2,973	16	111	14
50–64	2,952	15	144	18
≥ 65	1,870	10	51	6.5
**Sex**				
Female	9,637	50	352	45
Male	9,500	50	433	55
**Travel history**				
Yes^b^	NA		56	7.1
No	NA		601	77
Unknown	NA		128	16
**Vaccination status^c^**				
Not vaccinated	8,199	44	111	14
Started^d^	484	2.6	19	2.4
Vaccinated^e^	9,269	50	599	76
Booster vaccination^f^	597	3.2	56	7.1
**Previous SARS-CoV-2 infection confirmed by RT-PCR in Denmark** ^c,g^				
Yes	160	0.9	34	4.3
No	18,389	99	751	96
**Self-reported symptoms**				
Yes	NA		509	65
No	NA		157	20
Unknown	NA		119	15
**Hospitalisation**				
Yes	290	1.5	9	1.2
**Intensive care treatment**				
Yes	22	0.11	1	0.13
**Death**				
Yes	14	0.07	0	0

NA: not applicable; SARS-CoV-2: severe acute respiratory syndrome coronavirus 2.

^a^ Based on WGS-confirmed cases with sampling date from 22 November–7 December 2021.

^b^ South Africa (n = 16), Germany, Spain, Tanzania, Nigeria, Zimbabwe, United Kingdom, the Netherlands, Sweden and Belgium (sorted by frequency, only countries to which  ≥ 2 have reported travel is shown).

^c^ Based on SARS-CoV-2 Delta variant cases from TCDK only (n = 18,549).

^d^ One dose but not yet completed vaccination schedule (see footnote ^e^).

^e^ Vaccinated with two doses of Comirnaty (Pfizer/BioNTech, Mainz, Germany/ New York, United States), Vaxzevria (AstraZeneca, Cambridge, United Kingdom), Spikevax (Moderna, Cambridge, United States), or one dose of COVID-19 Vaccine Janssen (Johnson & Johnson, New Brunswick, United States) at least 14 days before positive test. By 9 December 2021, 76% of the Danish population had been fully vaccinated.

^f^ Booster vaccination ca 6 months after being fully vaccinated and at least 14 days before positive test.

^g^ Previous infection is defined as having a positive PCR tests more than 60 days from the current sample.

**Figure 2:**
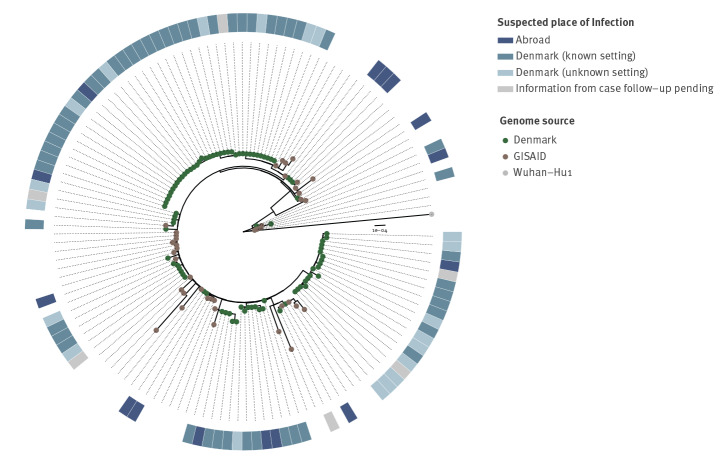
Maximum-likelihood phylogeny for consensus genomes from SARS-CoV-2 Omicron cases sequenced in Denmark as at 7 December 2021 (n = 92)

GISAID: Global initiative on sharing all influenza data; SARS-CoV-2: severe acute respiratory syndrome coronavirus 2.

As at 9 December, 644 cases had known information on place of infection (82% of all cases) and 56 of them (8.7%) reported a travel history. Sixteen reported travelling from South Africa, but also other African and European countries were reported (Table 2). These introductions are apparent on the phylogenetic tree (Figure 2). In addition, 464 cases (75%) reported a possible place of infection and 180 non-travellers (28%) did not report a suspicion on where they had been infected. To date, STPS has linked at least 83 Omicron cases to five large events in Denmark (> 100 participants). The number of cases related to these events is expected to increase as testing and contract tracing are ongoing. A high attack rate was reported at one of the events, a seasonal gathering with 150 participants, where 71 (47%) participants got infected. The SARS-CoV-2 Omicron variant was introduced to the gathering by a person who had been travelling to South Africa. This virus was further transmitted to three secondary schools and a concert with ca 2,000 participants in the same region, and subsequently to other events in other parts of Denmark. Furthermore, a hospital outbreak has been identified on a geriatric ward where at least four patients older than 80 years and two employees tested positive for Omicron. All of them were previously vaccinated.

Green nodes: Danish cases, showing suspected place of infection (heatmap); brown nodes: representative Omicron consensus genomes from the GISAID genome database not sequenced in Denmark (Supplementary Table S1), rooted on the Wuhan-Hu1 strain (grey node). Introductions are denoted in the heatmap as 'Abroad' [7-9].

## Ethical statement

This study did not require ethical approval as it is based on routine surveillance data on COVID-19.

## Discussion

Since the identification of the first Omicron case in Denmark, a steep increase in the number of cases has been observed. A major driver of this development was a large party with young adults – a population group with more social and close connections than adults and children. The rapid spread of the Omicron variant in Denmark is worrying, but not unexpected. On 2 December 2021, the European Centre for Disease Prevention and Control assessed a high probability of new introduction and community spread [10].

The response strategy in Denmark has been to delay transmission of the Omicron variant in order to gain time for roll-out of the third vaccine dose and the recently initiated vaccination programme for children aged 5 to 11 years. However, the rapid acceleration of cases catalysed by superspreading events challenged the mitigation. Despite the capacity to detect Omicron cases early, implementation of travel restrictions and implementation of extended contact tracing efforts, more than one in five cases cannot be linked to previous cases. This indicates that within 1.5 weeks from identifying the first case of Omicron, there is already widespread community transmission in Denmark, which challenges further epidemic control.

It is of concern that 83% of cases occurred in fully or booster-vaccinated people. Whether this observation is an artefact as the major superspreading events and subsequent chains of transmission have occurred primarily in young adults, and not yet spread to children, who have not been vaccinated, is still too early to say. The data are too immature to allow analysing the cases by vaccine type. However, it can be mentioned that 76% of the Danish population are fully vaccinated and 84% of these have received Comirnaty (Pfizer/BioNTech, Mainz, Germany/ New York, United States) followed by 14% with Spikevax (Moderna, Cambridge, United States). The composition of cases as well as the short follow-up period may affect the observed proportion of hospitalised cases. The proportion of asymptomatic cases should be interpreted considering the massive testing of contacts and relatively quick follow-up of cases, which means that SARS-CoV-2 Omicron cases may be caught early in their course of infection, potentially before the development of symptoms.

Of note, the earliest Omicron cases in Denmark occurred before South Africa announced the emergence of this variant. These cases reported travel history from Qatar and the Netherlands, indicating that the variant might have spread from the African continent before this. Also, later Danish travel-related cases are not only found among travellers returning from South Africa but also from other European countries, indicating that community spread is likely to be more widespread than reported.

Denmark has one of the highest RT-PCR testing capacities in the world and screens all positive RT-PCR tests with an Omicron-specific PCR [11]. Linking this information with national registers allows us to give a detailed overview of the early spread of the Omicron variant. We find several reasons for concern: (i) the rapid spread shortly after introduction despite extensive contact tracing efforts, (ii) the occurrence of several superspreading events with high attack rates and (iii) the high proportion of fully vaccinated Omicron cases. We observed nine hospitalisations and one case needed intensive care treatment. Owing to limited follow-up time and few admissions, it is too early to conclude on the severity of the Omicron variant.

## Conclusion

We show a rapid increase and spread of the SARS-CoV-2 Omicron variant in Denmark, a European country with high testing capacity, high vaccination coverage and limited natural immunity through SARS-CoV-2 infection. The introduction and spread occurred despite an early and comprehensive public health response. Spread was catalysed by superspreading events and challenges further epidemic control. Information from the earlier travel-related cases, with no travel history to Africa, suggests that community transmission is more widespread than reported. The high proportion of fully vaccinated Omicron cases is a concern, the implications are still being described. It is too early to draw conclusions on the severity of Omicron compared to previous SARS-CoV-2 variants, analyses on this are ongoing.

## Supplementary Data

534000 Supplement
