# Supplementary material for: Epidemiological characterisation of the first 785 SARS-CoV-2 Omicron variant cases in Denmark, December 2021
Source: Euro Surveill. 2021 Dec 16;26(50):2101146. doi: 10.2807/1560-7917.ES.2021.26.50.2101146 (PMC8728489; doi:10.2807/1560-7917.ES.2021.26.50.2101146)
Supplement: Supplement [file 21-01146_ESPENHAIN_Supplement.pdf]

Supplementary Material

This supplementary material is hosted by Eurosurveillance as supporting information alongside the article *Epidemiological characterisation of the first 785 SARS-CoV-2 Omicron variant cases in Denmark, December 2021*, on behalf of the authors, who remain responsible for the accuracy and appropriateness of the content. The same standards for ethics, copyright, attributions and permissions as for the article apply. Supplements are not edited by Eurosurveillance and the journal is not responsible for the maintenance of any links or email addresses provided therein.

We gratefully acknowledge the following Authors from the Originating laboratories responsible for obtaining the specimens, as well as the Submitting laboratories where the genome data were generated and shared via GISAID, on which this research is based.

All Submitters of data may be contacted directly via www.gisaid.org

Authors are sorted alphabetically.

Accession ID	Originating Laboratory	Submitting Laboratory	Authors
EPI_ISL_6914012, EPI_ISL_6914021, EPI_ISL_6914030, EPI_ISL_6914034	Ampath Laboratories	National Institute for Communicable Diseases of the National Health Laboratory Service	Amoako DG; Bhiman JN; Everatt J; Ismail A; Mahlangu B; Mnguni A; Mohale T; Ntuli N; Scheepers C; Wolter N
EPI_ISL_6774093	Botswana Harvard HIV Reference Laboratory	Botswana Harvard HIV Reference Laboratory	Boitumelo Zuze; Botshelo Radibe; Dorcas Maruapula; Joseph Makhema; Keoratile Ntshambiwa; Kgomotso Moruisi; Legodile Kooepile; Mosepele Mosepele; Mphaphi B. Mbulawa; Ontlametse T. Bareng; Pamela Smith-Lawrence; Roger Shapiro; Sefetogi Ramaologa; Shahin Lockman; Sikhulile Moyo; Simani Gaseitsiwe; Thongbotho Mphoyakgosi; Wonderful T. Choga
EPI_ISL_7263830	CAP Roger de Flor	Banc de Sang i Teixits	Carlos Hobeich; Francisco Vidal; Irene Corrales; Lorena Ramírez; Maria Glòria Soria; Natália Comes; Nina Borràs; Noemí Gonzalez; Silvia Sauleda
EPI_ISL_7154390	Clina-Lancet	National Reference Laboratory, Nigeria Centre for Disease Control	Catherine Okoi; Chimaobi Chukwu; Dr Ifedayo Adetifa; Dr Ndodo Nnaemeka; Dr Omoare Adesuyi; Nwando Mba; Olajumoke Babatunde; Olusola Anuoluwapo Akanbi; Oyeronke Ayansola
EPI_ISL_7266083	Clinical Microbiology Laboratory, Tel Aviv Sourasky Medical Center	Clinical Microbiology Laboratory, Tel Aviv Sourasky Medical Center	Alon Ziv; Amos Adler; Katya Levytskyi; Lior Handler; Ora Halutz
EPI_ISL_6951145	Color Genomics	Chiu Laboratory, University of California, San Francisco	Alicia Sotomayor-Gonzalez; Alicia Zhou; Amy Garlin; Charles Chiu; Darpun Sachdev; Katherine Hernandez; Scott Topper; Susan Philip; Venice Servellita; Yueyuan Zhang
EPI_ISL_6972689	Dept. of Laboratory Medicine	Dept. of Laboratory Medicine	Claudia Weber; Fabian Konig; Harald Esterbauer; Oswald Wagner; Robert Strassl; Sabina Plumer; Victoria Six
EPI_ISL_6841611	Dutch COVID-19 response team	National Institute for Public Health and the Environment (RIVM)	Adam Meijer; AnneMarie van den Brandt; Annelies Kroneman; Bas van der Veer; Chantal Reusken; Dennis Schmitz; Dirk Eggink; Florian Zwagemaker; Harry Vennema; Ivo van Walle; Jeroen Cremer; Karim Hajji; Kim Freriks; Linda van Someren; Lisa Wijsman; Lynn Aarts; Ryanne Jaarsma; Sanne Bos; Sharon van den Brink; Stijn van Rossum; on behalf of the national COVID-19 response team
EPI_ISL_6989155	Furst Medical Laboratory	Norwegian Institute of Public Health, Department of Virology	Atiya R Ali; Debech Nadia; Engebretsen Serina Beate; Garcia Llorente Ignacio; Hilde Elshaug; Hilde Vollan; Jon Bråte; Kamilla Heddeland Instefjord; Karoline Bragstad; Kathrine Stene-Johansen; Line Victoria Moen; Marie Paulsen Madsen; Olav Hungnes; Pedersen Benedikte Nevjen; Rasmus Riis Kopperud
EPI_ISL_7313494	GH A.CHENEVIER-H.MONDOR	Department of Virology, Henri Mondor University Hospital, Assistance Publique Hôpitaux de Paris, Université Paris-Est Créteil, INSERM U955	Alexandre Soulier; Christophe Rodriguez; Elisabeth Trawinski; Guillaume Gricourt; Jean-Michel Pawlotsky; Melissa N'Debi; Slim Fourati; Vanessa Demontant
EPI_ISL_6892683, EPI_ISL_6892693	Germano de Sousa	Instituto Nacional de Saude (INSA)	Borges et al
EPI_ISL_6900139, EPI_ISL_6900143	HELEN JOSEPH LABORATORY	National Institute for Communicable Diseases of the National Health Laboratory Service	Amoako DG; Bhiman JN; Everatt J; Ismail A; Mahlangu B; Mnguni A; Mohale T; Ntuli N; Scheepers C
EPI_ISL_6832108	Home Quarantine Taskforce	Hong Kong Department of Health	Alan K.L. Tsang; Edman T.K. Lam; Ken H.L. Ng; Peter C.W. Yip; Rickjason C.W. Chan
EPI_ISL_7156753	IHU Mediterranee Infection	IHU Mediterranee Infection	Philippe Colson et al.
EPI_ISL_6959868	Institute for Medical Virology, Frankfurt	Institute for Medical Virology, Frankfurt	Ciesek S.; Toptan T.
EPI_ISL_6927893, EPI_ISL_7299709	Lighthouse Lab in Glasgow	Wellcome Sanger Institute for the COVID-19 Genomics UK (COG-UK) Consortium	Anna Dominiczak and Alex Alderton; Carol Clugston; Cordelia Langford; David Gray; David K. Jackson; Dominic Kwiatkowski; Ewan Harrison; Harper VanSteenhouse; Ian Johnston; Jeffrey Barrett; John Sillitoe on behalf of the Wellcome Sanger Institute COVID-19 Surveillance Team; Roberto Amato; Sonia Goncalves; Yumi Kasai
EPI_ISL_7023675, EPI_ISL_7023726, EPI_ISL_7147199, EPI_ISL_7198902, EPI_ISL_7203185, EPI_ISL_7300986	Lighthouse Lab in Milton Keynes	Wellcome Sanger Institute for the COVID-19 Genomics UK (COG-UK) Consortium	Cordelia Langford; David K. Jackson; Dominic Kwiatkowski; Ewan Harrison; Ian Johnston; Jeffrey Barrett; John Sillitoe on behalf of the Wellcome Sanger Institute COVID-19 Surveillance Team; Roberto Amato; Sonia Goncalves; The Lighthouse Lab in Milton Keynes and Alex Alderton
EPI_ISL_6795189, EPI_ISL_6795190	National Health Laboratory Services, Virology	CERI, Centre for Epidemic Response and Innvoation, Stellenbosch University and KRISP, KZN Research Innovation and Sequencing Platform, UKZN.	Arisha Maharaj; Florette Treurnicht; Giandhari J; Kathleen Subramoney; Naidoo Y; Pillay S; Ramphal U; Ramphal Y; San JE; Tegally H; Tshiabuila D; Wilkinson E; de Oliveira T
EPI_ISL_6810484	National Health Laboratory Services, Virology, Charlotte Maxeke Johannesburg hospital, Parktown, Johannesburg, Gauteng	CERI, Centre for Epidemic Response and Innvoation, Stellenbosch University and KRISP, KZN Research Innovation and Sequencing Platform, UKZN.	Amoaka D; Arisha Maharaj; Avani Bharuthram; Bester P; Bhiman J; Engelbrecht S; Everatt J; Florette Treurnicht; Goedhals D; Hardie D; Hsiao M; Iranzadeh A; Kathleen Subramoney; Lessells R; Makatini Z; Maponga T; Mdlalose N; Mlisana K; Moir M; NGS-SA (Scheepers C; Naidoo Y; Nkhensani Mtileni; Nyaga M) Giandhari J; Oluwakemi M; Pillay S; Preiser W; Ramphal U; Ramphal Y; San JE; Tegally H; Tshiabuila D; Venter M; Wilkinson E; Williamson C; de Oliveira T; von Gottberg A
EPI_ISL_6939067	National Influenza Centre	National Influenza Centre	; Benjamiin B. Lindsey; Benjamin H. Foulkes; Bless Seyram Agbenyo; Bright Adu; Ernest Asiedu; Franklin Asiedu-Bekoe; Hilda Opoku Frempong; Ivy A. Asante; Joseph Oliver-Commey; Joyce Appiah-Kubi; Keren Okyerebea Attiku; Linda Boatemaa; Lorreta Kwasah; Mathew D. Parker; Michael Marks; Mildred Adusei-Poku; Quaneeta Mohktar; Sharon Hsu; Thushan I de Silva; William K. Ampofo
EPI_ISL_6842158, EPI_ISL_6842166	PathCare, Cape Town	Division of Medical Virology, National Health Laboratory Service (NHLS), Tygerberg Hospital / Stellenbosch University	Gert van Zyl; Jean Maritz; Kamela Mahlakwane; Nadine Cronje; Petra Raimond; Shannon Wilson; Tania Stander; Tongai Maponga; Wolfgang Preiser
EPI_ISL_7265233	St Vincent's Pathology (SydPath)	NSW Health Pathology - Institute of Clinical Pathology and Medical Research; Westmead Hospital; University of Sydney	Arnott A.; Draper J.; Gall M.; Martinez E.; Rockett R.; Sintchenko V.; on behalf of ICPMR
EPI_ISL_6795840, EPI_ISL_7015173, EPI_ISL_7015196, EPI_ISL_7015197	ZARV/NHLS, Department Medical Virology, University of Pretoria	CERI, Centre for Epidemic Response and Innvoation, Stellenbosch University and KRISP, KZN Research Innovation and Sequencing Platform, UKZN.	Adriano Mendes; Amoaka D; Amy Strydom; Arisha Maharaj; Bester P; Bhiman J; Engelbrecht S; Everatt J; Giandhari J; Goedhals D; Hardie D; Hsiao M; Iranzadeh A; Lessells R; Makatini Z; Maponga T; Mdlalose N; Micheala Davids; Mlisana K; Moir M; NGS-SA (Scheepers C; Naidoo Y; Nyaga M) Giandhari J; Oluwakemi M; Pillay S; Preiser W; Ramphal U; Ramphal Y; San JE; Sim Mayaphi and Marietjie Venter; Tegally H; Tshiabuila D; Venter M; Wilkinson E; Williamson C; de Oliveira T; von Gottberg A
